# Cancer Risk in Children and Adolescents with Birth Defects: A Population-Based Cohort Study

**DOI:** 10.1371/journal.pone.0069077

**Published:** 2013-07-17

**Authors:** Lorenzo D. Botto, Timothy Flood, Julian Little, Mark N. Fluchel, Sergey Krikov, Marcia L. Feldkamp, Yuan Wu, Rhinda Goedken, Soman Puzhankara, Paul A. Romitti

**Affiliations:** 1 Division of Medical Genetics, Department of Pediatrics, University of Utah, Salt Lake City, Utah, United States of America; 2 Arizona Department of Health Services, Phoenix, Arizona, United States of America; 3 Department of Epidemiology and Community Medicine, University of Ottawa, Ontario, Canada; 4 Division of Pediatric Hematology and Oncology, Department of Pediatrics, University of Utah, Salt Lake City, Utah, United States of America; 5 Department of Epidemiology, College of Public Health, The University of Iowa, Iowa City, Iowa, United States of America; National Taiwan University Hospital, Taiwan

## Abstract

**Objective:**

Birth defects are an increasing health priority worldwide, and the subject of a major 2010 World Health Assembly Resolution. Excess cancer risk may be an added burden in this vulnerable group of children, but studies to date have provided inconsistent findings. This study assessed the risk for cancer in children and young adolescents with major birth defects.

**Methods and Findings:**

This retrospective, statewide, population-based, cohort study was conducted in three US states (Utah, Arizona, Iowa). A cohort of 44,151 children and young adolescents (0 through 14 years of age) with selected major, non-chromosomal birth defects or chromosomal anomalies was compared to a reference cohort of 147,940 children without birth defects randomly sampled from each state’s births and frequency matched by year of birth. The primary outcome was rate of cancer prior to age 15 years, by type of cancer and type of birth defect. The incidence of cancer was increased 2.9-fold (95% CI, 2.3 to 3.7) in children with birth defects (123 cases of cancer) compared to the reference cohort; the incidence rates were 33.8 and 11.7 per 100,000 person-years, respectively. However, the excess risk varied markedly by type of birth defect. Increased risks were seen in children with microcephaly, cleft palate, and selected eye, cardiac, and renal defects. Cancer risk was not increased with many common birth defects, including hypospadias, cleft lip with or without cleft palate, or hydrocephalus.

**Conclusion:**

Children with some structural, non-chromosomal birth defects, but not others, have a moderately increased risk for childhood cancer. Information on such selective risk can promote more effective clinical evaluation, counseling, and research.

## Introduction

An estimated 3% of babies are born with major birth defects [1]–[3]. In the United States, this translates into at least 120,000 newly affected babies every year, and millions more worldwide. The impact of birth defects is profound and wide-ranging, in affluent as well as, increasingly, in lower income countries. Recognizing such impact, the World Health Organization has recently identified birth defect prevention and care as a global priority [4].

The health burden associated with birth defects is significant; in the United States and many other countries, birth defects are the leading cause of infant death and a major contributor to disability and pediatric hospitalizations [3], [5], [6]. Cancer risk could add to this recognized burden. However, whereas the evidence for increased cancer risk is robust for conditions such as Down syndrome [7]–[13], it appears less consistent for many structural birth defects, with studies providing varying estimates for different types of birth defects and often only for broad categories with limited clinical specificity [7]–[19].

Research has uncovered the basis for a few associations between birth defects and cancer, mostly related to genes involved in growth homeostasis [20] and DNA repair/maintenance [20], [21]. However, for most cases in which birth defects and cancer occur together, the underlying causes and mechanisms, whether genetic or environmental, remain unclear. Further clues could be uncovered by better information on patterns and magnitude of cancer risk in children with birth defects. By helping to identify who is, and who is not, at increased cancer risk, such information could translate into better care and long term outcomes.

However, such studies are challenging: they require large population-based samples, longitudinal follow-up, detailed and validated information on clinical phenotype, and appropriate reference populations. In this study, we evaluated a large population-based cohort of U.S. children and young adolescents with birth defects to quantify and qualify their risk of cancer prior to age 15 years.

## Methods

### Study Design, Setting, Subjects (Table 1)

The Institutional Review Board (IRB) in each of the three states (University of Utah, IRB; Utah Department of Health, IRB, Resource for Genetic and Epidemiologic Research Review Board; Arizona Department of Health Service, Human Subjects Review Board; The University of Iowa, Human Subjects Research Office) approved the study. These IRBs waived the requirement to obtain informed consent because the study used existing data collected under public health surveillance statutes as reportable conditions and required no patient contact.

The three states partnered to increase the available study population. This partnership was facilitated by the presence of similar population-based surveillance programs of birth defects and of cancer (Table 1) with access to clinical abstracts for clinical case review. The study compared two population-based cohorts derived from an underlying birth population of 2.7 million singleton liveborn infants of resident mothers in Utah, Arizona, and Iowa (“UTAZIA” study). The index cohort included all children with selected major, non-chromosomal birth defects and selected chromosomal anomalies identified from these states’ population-based birth defect surveillance programs. The reference cohort consisted of children without birth defects, randomly selected from the states’ birth certificates and frequency-matched (3∶1 ratio) to the index cohort by year of birth. We then linked the reference cohort to the data in the birth defect surveillance programs to exclude children with birth defects.

**Table 1:** Cohorts, follow-up time, and surveillance programs, UTAZIA study.

	Arizona	Iowa	Utah	Total
Birth years	1986 to 2004	1983 to 2004	1994 to 2006	1983 to 2006
Follow-up*	15 years	15 years	15 years	15 years
Total liveborn infants	1,328,053	847,258	604,126	2,779,437
Index cohort (with birth defects)	19,629	16,463	8,059	44,151
Person Years of follow-up	165,607	151,052	46,999	363,659
Median follow-up (years)	10.1	11.3	6.2	9.2
Reference cohort	66,503	53,743	27,694	147,940
Person Years of follow-up	646,842	551,817	181,576	1,380,235
Median follow-up (years)	10.3	11.6	6.3	9.3
Ascertainment basis	Population-based	Population-based	Population-based	
Geographic coverage	State-wide	State-wide	State-wide	
Case ascertainment	Active	Active	Active	
Age cutoff at first diagnosis	12 months	12 months	24 months	
Age cutoff for additional defects	Any age	Any age	Any age	
Clinical review of cases	Yes(Obstetrician-gynecologist)	Yes (Geneticist)	Yes(Geneticist)	
Cancer registry†	NPCR	SEER	SEER	

UTAZIA: Utah, Arizona, Iowa.

* Follow-up was from birth up to but excluding the 15^th^ birthday.

† NPCR, National Program of Cancer Registries; SEER, Surveillance, Epidemiology and End Results program.

### Data Sources, Inclusions, Exclusions, Clinical Case Review

**Figure 1:**
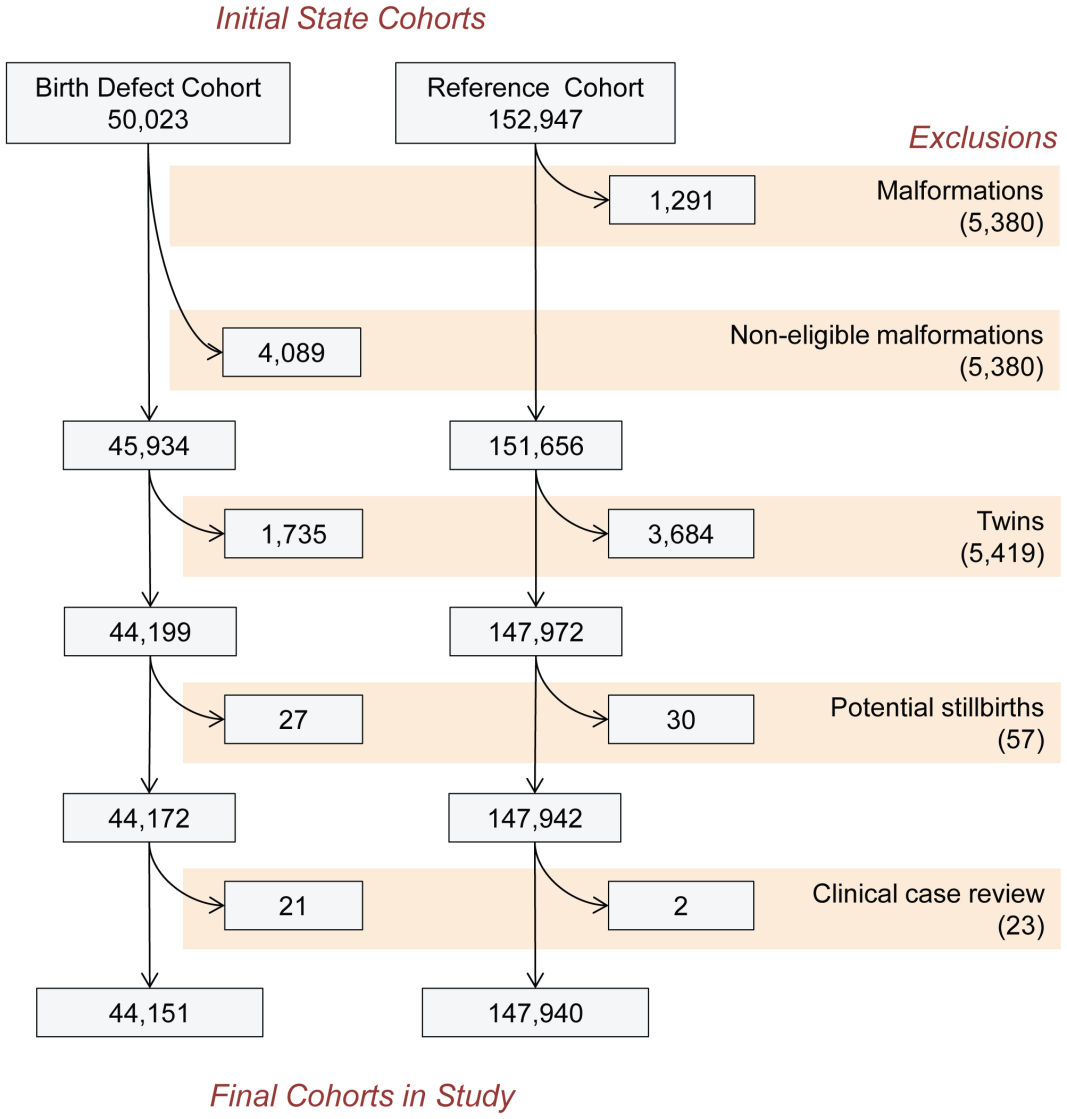
Inclusions and exclusions leading to final cohorts in the UTAZIA study of birth defects and cancer.

The primary data sources were the birth surveillance programs, to select the birth defect cohort, and vital records databases, to select the reference cohort and to obtain death abstracts. These initial cohorts were reviewed (Figure 1) for inclusion and exclusion criteria, leading to the final study cohorts. Briefly, subjects were selected based on the presence of selected major birth defects, as defined by the National Birth Defect Prevention Network [22]. These birth defects account for most clinically significant conditions (found in 1.5 to 2 percent of births overall) while excluding common conditions that are inconsistently identified around the time of birth, may resolve spontaneously, or require minor or no medical procedures (e.g., patent ductus arteriosus in the newborn, bicuspid aortic valve without stenosis, undescended testis, pre-auricular tags, pigmented nevi).

Because of early lethality, we also excluded infants with anencephaly and babies weighing less than 400g at birth or less than 20 weeks of gestational age. Clinical case review by a board-certified clinical geneticist and a pediatric oncologist led to the exclusion of secondary conditions (19 in the birth defect cohort) misclassified as primary birth defects (e.g., “hydrocephalus” due to brain tumors obstructing the flow of cerebrospinal fluid [8], hydronephrosis due to mass effect of abdominal tumors, and “anophthalmia” due to removal of the eye following a diagnosis of retinoblastoma), and also to the exclusion of all cases of Langerhans cell histiocytosis (two in the reference cohort, one in the birth defect cohort) because of the controversy over whether this condition is a true malignancy [23], [24]. The final study cohorts are summarized in Table 1.

### Data Classification

We classified structural, non-chromosomal birth defects based on clinical and developmental considerations. For example, we separated limb deficiencies into transverse, preaxial, and postaxial types. We also classified heart defects hierarchically [25]: every child with a heart defect was assigned to a single heart defect group, based on embryologic timing and mechanism [25]–[27]. Complex heart defects included single ventricle and its variants (e.g., double inlet left ventricle) and laterality defects (e.g., situs ambiguus). When a non-chromosomal birth defect case had two or more unrelated major birth defects, it was counted in each specific birth defect group; for example, a case of spina bifida and hypospadias contributed to the counts for both spina bifida and hypospadias. However, group totals, overall and by organ system (e.g., non-chromosomal birth defects, brain defects) count people with these conditions, not defects.

We included children with trisomy 21 plus Klinefelter syndrome (48 XXY, +21) in the trisomy 21 group. We classified children with other chromosomal anomalies (e.g., deletion 22q11) who had a major birth defect (e.g., a heart defect) in “other chromosomal anomalies.” We grouped cancers using the International Classification of Childhood Cancers [28]. One child developed two primary cancers; we considered only the first diagnosed cancer.

### Statistical Methods

We restricted the analysis of individual structural birth defects to non-chromosomal cases. We analyzed chromosomal cases separately, by chromosomal diagnosis. To identify cases of cancer, we linked both cohorts to each state’s cancer surveillance program. To identify deaths, we also linked these cohorts by name, birth date, and sex to administrative databases (state vital record, National Death Index, and Social Security Administration). Linkages used a combined probabilistic and deterministic approach, followed by manual review of individual record matches [29]. The follow-up time (Table1) was 363,000 person-years (PY) in the index cohort and 1.38 million PY in the reference cohort.

Using these data we computed person-time as the duration from birth to cancer diagnosis prior to age 15 years, death, or to the end of follow-up period (December 2005 for Arizona and Iowa, and December 2008 for Utah), whichever came first. Age-specific cancer incidence was based on the number of cases per 100,000 PY of follow-up through that age. To appropriately account for censored data, we used the Kaplan-Meier method to estimate cancer-free survival and cumulative cancer incidence. We used Cox proportional hazards models to adjust for potential confounders, including preterm birth, birth weight, sex, and maternal race/ethnicity, age, education, and state. Missing data were not imputed. Due to confidentiality agreements, the number of cases of cancer is not specified if it is fewer than five. Sample size of the cohort with birth defects was determined based on the largest number available from the three birth defect surveillance programs. Statistical analyses were done with SAS 9.1 (SAS Institute, Cary, NC).

## Results

Baseline characteristics of the cohort with birth defects and the reference cohort are shown in Table 2. The cohort with birth defects had a higher proportion of male births, shorter length of pregnancies, and lower average birth weight. The male excess was driven by the presence of hypospadias, a common male-specific defect. The reference cohort was similar to the total underlying birth population of 2.7 million live births for the same years (data not shown) for all the maternal and child characteristics available in birth certificates. Cancer incidence was 2.9-fold higher in the index cohort (33.8 per 100,000 PY) compared to the reference cohort (11.7 per 100,000 PY), with considerable variation by phenotype (Table 3). Because adjustment for maternal and child characteristics did not appreciably change the estimates, we present the unadjusted estimates and summarize the adjusted estimates in Table S1.

**Table 2:** Maternal and child characteristics in index and reference cohorts, UTAZIA study.

		Index Cohort (with birth defects)		Reference Cohort (without birth defects)	
		No.	%	No.	%
**Cohort size**		44,151		147,940	
**Maternal age (years)**					
less than 20 years		5,000	11.3	15,971	10.8
20 to 24		11,752	26.6	39,815	26.9
25 to 29		11,947	27.1	42,740	28.9
30 to 34		8,052	18.2	27,816	18.8
35 and over		5,006	11.3	13,264	9.0
Missing		2,394	5.4	8,334	5.6
**Maternal race/ethnicity**					
Non-Hispanic White		32,655	74.0	107,061	72.4
Non-Hispanic Black		993	2.2	3,806	2.6
Hispanic		6,532	14.8	23,610	16.0
Native American		1,922	4.4	6,020	4.1
Asian/Pacific Islander		1,441	3.3	5,913	4.0
Other		132	0.3	549	0.4
Missing		476	1.1	981	0.7
**Maternal education**					
High School or less		24,308	55.1	78,880	53.3
More than High School		19,466	44.1	68,015	46.0
Missing		377	0.9	1,045	0.7
**Child Sex**					
Male		28,692	65.0	75,318	50.9
Female		15,436	35.0	72,622	49.1
Ambiguous/missing		23	0.0	0	0.0
**Child birth weight**					
Less than 2500 g		7,756	17.6	7,141	4.8
2500–3999 g		32,625	73.9	125,326	84.7
4000 and more g		3,595	8.1	15,345	10.4
Missing		175	0.4	128	0.1
**Child gestational age**					
Less than 37 weeks		7,998	18.1	10,014	6.8
37 or more weeks		31,458	71.3	116,712	78.9
Missing		4,695	10.6	21,214	14.3

UTAZIA: Utah, Arizona, Iowa.

Note: percentages may not add up to 100 due to rounding.

**Table 3:** Risk for cancer by type of chromosomal anomaly and major birth defect, UTAZIA study.

Diagnosis		Cohort size	Person Years follow-up	Cases of cancer	Incidence rate*	Incidence rate ratio		95% CI
**Cohort without Birth Defects (Reference)**		147,940	1,380,235	161	11.7	1.0		Reference
**Cohort with Birth Defects**		44,151	363,659	123	33.8	2.9	†	2.3–3.7
** Birth Defects, non-chromosomal**		39,726	333,782	77	23.1	2.0	†	1.5–2.6
Brain defects		4,311	34,813	9	25.9	2.2	†	1.1–4.3
Neural tube defects, all		1,334	11,056	<5	27.1	2.3		0.7–7.3
Spina bifida w/out anencephalus		1,108	9,588	<5	31.3	2.7		0.9–8.4
Encephalocele		226	1,468	0	0.0	0.0		
Microcephaly		1,801	14,611	5	34.2	2.9	†	1.2–7.1
Holoprosencephaly		63	349	<5	286.3	24.5	†	3.4–175.3
Hydrocephalus (no spina bifida)		1,271	9,780	<5	10.2	0.9		0.1–6.3
Eye defects		928	7,305	8	109.5	9.4	†	4.6–19.1
Anophthalmia/microphthalmia		432	3,064	<5	97.9	8.4	†	2.7–26.3
Congenital cataract		530	4,453	6	134.8	11.6	†	5.1–26.1
Aniridia		31	278	<5	359.2	30.8	†	4.3–219.9
Ear defects (anotia/microtia)		626	5,162	<5	19.4	1.7		0.2–11.9
Craniosynostosis		422	2,509	<5	39.9	3.4		0.5–24.4
Heart defects		11,211	82,890	28	33.8	2.9	†	1.9–4.3
Complex heart defects		465	2,390	<5	41.8	3.6		0.5–25.6
Common truncus		172	656	<5	152.3	13.1	†	1.8–93.3
Transposition of great arteries		687	4,627	<5	43.2	3.7		0.9–14.9
Tetralogy of Fallot		759	5,359	<5	18.7	1.6		0.2–11.4
Atrioventricular septal defect (AV canal)		350	1,890	<5	105.8	9.1	†	2.2–36.6
Total anomalous pulmonary venous return		64	418	<5	239.1	20.5	†	2.9–146.4
Pulmonary valve atresia		182	985	0	0.0	0.0		
Tricuspid valve atresia and stenosis		153	1,200	0	0.0	0.0		
Ebstein anomaly		141	967	0	0.0	0.0		
Hypoplastic left heart syndrome		509	872	0	0.0	0.0		
Coarctation of the aorta		1,024	8,032	<5	12.5	1.1		0.1–7.6
Aortic valve stenosis		540	3,821	<5	52.3	4.5	†	1.1–18.1
Other major congenital heart defects		203	1,638	0	0.0	0.0		
Pulmonary valve stenosis		1,037	7,760	<5	25.8	2.2		0.5–8.9
Ventricular septal defect, membranous		2,330	17,492	5	28.6	2.5	†	1.0–6.0
Ventricular septal defect, NOS		1,276	15,257	<5	26.2	2.2		0.8–6.1
Atrial septal defect		1,404	10,157	6	59.1	5.1	†	2.2–11.4
Orofacial clefts		4,756	41,001	6	14.6	1.3		0.6–2.8
Cleft palate, without cleft lip		1,656	13,273	5	37.7	3.2	†	1.3–7.9
Cleft lip with or without cleft palate		3,100	27,728	<5	3.6	0.3		0–2.2
Choanal atresia		387	3,121	0	0.0	0.0		
Gastrointestinal (GI) defects		7,207	65,278	13	19.9	1.7	†	1.0–3.0
GI atresias, all		1,367	10,649	<5	28.2	2.4		0.8–7.6
Esophageal atresia/ TE Fistula		562	4,364	<5	22.9	2.0		0.3–14
Duodenal atresia		67	410	0	0.0	0.0		
Jejunal/Ileal atresia		79	508	0	0.0	0.0		
Small Intestinal atresia		9	72	0	0.0	0.0		
Rectal/intestinal atresia/stenosis		1,025	8,020	<5	37.4	3.2	†	1.0–10
Pyloric stenosis		5,071	48,431	6	12.4	1.1		0.5–2.4
Hirschsprung disease		377	3,317	<5	60.3	5.2	†	1.3–20.8
Biliary atresia		163	1,128	<5	177.3	15.2	†	3.8–61.3
Abdominal wall defects and variants		1,479	11,258	<5	26.6	2.3		0.7–7.2
Omphalocele		368	2,681	<5	37.3	3.2		0.4–22.8
Gastroschisis		972	7,442	<5	26.9	2.3		0.6–9.3
Cloacal exstrophy		17	102	0	0.0	0.0		
Bladder exstrophy		66	475	0	0.0	0.0		
Epispadias		131	1,155	0	0.0	0.0		
Diaphragmatic hernia		605	3,220	<5	62.1	5.3	†	1.3–21.5
Genitourinary (GU) defects		10,887	92,735	20	21.6	1.8	†	1.2–2.9
Renal, all		4,349	31,308	12	38.3	3.3	†	1.8–5.9
Renal agenesis/hypoplasia		977	5,168	5	96.7	8.3	†	3.4–20.2
	Obstructive GU defect	3,561	27,226	8	29.4	2.5	†	1.2–5.1
Hypospadias (includes 1st degree)		6,691	62,574	8	12.8	1.1		0.5–2.2
Limb deficiencies		1,019	8,987	<5	22.3	1.9		0.5–7.7
Transverse		663	5,669	<5	35.3	3.0		0.7–12.2
Preaxial		299	2,169	<5	46.1	4.0		0.6–28.2
Postaxial		332	2,770	0	0.0	0.0		
Limb deficiency, NEC/NOS		439	4,038	0	0.0	0.0		
Amniotic bands		218	1,740	0	0.0	0.0		
** Chromosomal anomalies**		4,425	29,877	46	154.0	13.2	†	9.5–18.3
Trisomy 21 (Down syndrome)		3,202	25,876	43	166.2	14.2	†	10.2–19.9
Trisomy 18		378	237	0	0.0	0.0		
Trisomy 13		237	178	0	0.0	0.0		
Other chromosomal conditions		608	3,587	<5	83.6	7.2	†	2.3–22.5

UTAZIA: Utah, Arizona, Iowa.

* Rates are per 100,000 Person Years.

† *P* < 0.05 for incidence rate ratios (vs. reference cohort).

NOS, not otherwise specified; NEC, not elsewhere classified; TE fistula, tracheosophageal fistula.

### Non-chromosomal Birth Defects

Compared to the reference cohort, cancer risk was increased two-fold among children with non-chromosomal birth defects. Table 3 summarizes cancer risk by type of birth defect. Among defect groups with at least three cases of cancer, risk was increased in children with eye defects, microcephaly, cleft palate, and some heart and renal defects. Retinoblastoma occurred in a small number of children with cataracts and microphthalmia (data not shown). We also identified a positive association between heart defects as a group and cancer; however, subgroup analysis was limited by the small number of cancer diagnoses by type of heart defect. The types of cancer for which risk was increased included the “embryonal” childhood cancers, such as neuroblastoma, retinoblastoma, and hepatoblastoma (Table 4). In contrast, for many common defects, including hypospadias, pyloric stenosis, cleft lip with or without cleft palate, and hydrocephalus, cancer risk was not increased compared to the reference cohort.

**Table 4:** Risk for specific types of cancer in children with structural birth defects, UTAZIA study. Chromosomal conditions were excluded.

Cancer Type	Cohort without Birth Defects (Reference)		Cohort with Birth Defects				
	N = 147,940		N = 44,151				
	No. with Cancer	Rate*	No. with Cancer	Rate*	Incidence Rate Ratio		95% CI
Leukemia	45	3.3	13	3.9	1.19		0.6–2.2
Acute lymphoid leukemia	36	2.6	12	3.6	1.38		0.7–2.6
Acute myeloid leukemia	5	0.4	<5	0.3	0.83		0.1–7.1
Other/unspecified leukemia	<5	0.3	0	0.0	0.00		
Myelodysplastic/ myeloproliferative disease	<5	0.1	<5	0.9	12.41	‡	1.3–119.3
Lymphoma	10	0.7	<5	1.2	1.65		0.5–5.3
Non-Hodgkin lymphoma	6	0.4	<5	0.3	0.69		0.1–5.7
Hodgkin lymphoma	<5	0.3	<5	0.3	1.03		0.1–9.2
Lymphoma, not specified	0	0.0	<5	0.6	–		
Brain tumor	38	2.8	16	4.8	1.74		1.0–3.1
Astrocytoma	17	1.2	5	1.5	1.22		0.4–3.3
Medulloblastoma	13	0.9	<5	1.2	1.27		0.4–3.9
Other brain tumor	8	0.6	7	2.1	3.62	†	1.3–10.0
Neuroblastoma spectrum	17	1.2	11	3.3	2.68	†	1.3–5.7
Neuroblastoma	15	1.1	8	2.4	2.21		0.9–5.2
Other peripheral nervous system tumor	<5	0.1	<5	0.9	6.20	†	1–37.1
Retinoblastoma	7	0.5	6	1.8	3.54	†	1.2–10.5
Kidney tumor	16	1.2	<5	1.2	1.03		0.3–3.1
Wilms tumor	15	1.1	<5	1.2	1.10		0.4–3.3
Other kidney tumor	<5	0.1	0	0.0	0.00		
Liver tumor	<5	0.1	8	2.4	16.54	§	3.5–77.9
Hepatoblastoma	<5	0.1	7	2.1	14.47	§	3.0–69.7
Other liver tumors	0	0.0	<5	0.3	–		
Sarcoma	16	1.2	6	1.8	1.55		0.6–4.0
Rhabdomyosarcoma	5	0.4	<5	1.2	3.31		0.9–12.3
Osteosarcoma	<5	0.1	0	0.0	0.00		
Ewing sarcoma	<5	0.1	<5	0.3	2.07		0.2–22.8
Fibrosarcoma	<5	0.3	0	0.0	0.00		
Other soft tissue sarcoma	<5	0.2	<5	0.3	1.38		0.1–13.8
Germ cell, trophoblastic, and gonadal tumor	6	0.4	6	1.8	4.14	†	1.3–12.8
Teratoma	<5	0.2	<5	1.2	5.51	†	1.2–24.6
Other germ cell tumor	<5	0.2	<5	0.6	2.76		0.5–16.5
Miscellaneous tumors	<5	0.2	0	0.0	0.00		
**Total**	**161**	**11.7**	**77**	**23.1**	**1.98**	§	**1.5–2.6**
Tumors other than leukemias and lymphomas	105	7.6	57	17.1	2.2	§	1.6–3.1

UTAZIA: Utah, Arizona, Iowa.

* Rates are per 100,000 Person Years.

† *P* < 0.05;

‡ *P* < 0.01;

§ *P* < 0.001 for incidence rate ratios vs. reference cohort.

### Chromosomal Anomalies

No case of cancer was reported among 615 children with trisomy 13 or 18 (Table 3). Among children with Down syndrome, cancer incidence was 14-fold higher than in the reference cohort. Children with Down syndrome represented 7% of the index cohort (3202/44151) but contributed 35% of the cancer diagnoses (43/123). The distribution of common vs. variant forms of Down syndrome (e.g., Robertsonian (21;21) translocation, partial duplication of chromosome 21, or trisomy 21 with Klinefelter syndrome) were similar in children with Down syndrome who did vs. did not develop cancer. The excess cancer risk in children with Down syndrome was driven mainly by leukemias (Table 5), with the highest incidence rate ratio (IRR) observed for acute myeloid leukemia (IRR, 224.0). As a group, cancers other than leukemias and lymphomas (Table 5, bottom row) occurred at a frequency comparable to the reference cohort (IRR, 1.0), although within this group the rates of individual types of cancer varied considerably, with increased rates noted for myelodysplastic disorders, osteosarcoma, and teratoma (Table 5).

**Table 5:** Risk for specific types of cancer in children with Down syndrome (trisomy 21), UTAZIA study.

Cancer Type	Cohort without Birth Defects (Reference)		Cohort with Down Syndrome				
	N = 147,940		N = 3,202				
	No. with Cancer	Rate*	No. with Cancer	Rate*	Incidence Rate Ratio		95% CI
Leukemia	45	3.3	38	146.9	45.0	§	29.2–69.4
ALL (Acute lymphoid leukemia)	36	2.6	16	61.8	23.7	§	13.2–42.7
AML (Acute myeloid leukemia)	5	0.4	21	81.2	224.0	§	84.5–594.1
Other/unspecified leukemia	<5	0.3	<5	3.9	13.3	‡	1.5–119.3
Myelodysplastic/ myeloproliferative disease	<5	0.1	<5	7.7	106.7	§	9.7–1,176.6
Lymphoma	10	0.7	<5	3.9	5.3		0.7–41.7
Non-Hodgkin lymphoma	6	0.4	0	0.0	0.0		
Hodgkin lymphoma	<5	0.3	0	0.0	0.0		
Lymphoma, not specified	0	0.0	<5	3.9			
Brain tumor	38	2.8	0	0.0	0.0		
Astrocytoma	17	1.2	0	0.0	0.0		
Medulloblastoma	13	0.9	0	0.0	0.0		
Other brain tumor	8	0.6	0	0.0	0.0		
Neuroblastoma spectrum	17	1.2	0	0.0	0.0		
Neuroblastoma	15	1.1	0	0.0	0.0		
Other peripheral nervous system tumor	<5	0.1	0	0.0	0.0		
Retinoblastoma	7	0.5	0	0.0	0.0		
Kidney tumor	16	1.2	0	0.0	0.0		
Wilms tumor	15	1.1	0	0.0	0.0		
Other kidney tumor	<5	0.1	0	0.0	0.0		
Liver tumor	<5	0.1	0	0.0	0.0		
Hepatoblastoma	<5	0.1	0	0.0	0.0		
Other liver tumors	0	0.0	0	0.0	0.0		
Sarcoma	16	1.2	<5	3.9	3.3		0.4–25.1
Rhabdomyosarcoma	5	0.4	0	0.0	0.0		
Osteosarcoma	<5	0.1	<5	3.9	26.7	§	2.4–294.1
Ewing sarcoma	<5	0.1	0	0.0	0.0		
Fibrosarcoma	<5	0.3	0	0.0	0.0		
Other soft tissue sarcoma	<5	0.2	0	0.0	0.0		
Germ cell, trophoblastic, and gonadal tumor	6	0.4	<5	3.9	8.9	†	1.1–73.8
Teratoma	<5	0.2	<5	3.9	17.8	§	1.8–170.9
Other germ cell tumor	<5	0.2	0	0.0	0.0		
Miscellaneous tumors	<5	0.2	0	0.0	0.0		
**Total**	**161**	**11.7**	**43**	**166.2**	**14.2**	§	**10.2–19.9**
Tumors other than leukemias and lymphomas	105	7.6	<5	7.9	1.0		0.2–3.9

UTAZIA: Utah, Arizona, Iowa.

* Rates are per 100,000 Person Years.

† *P* < 0.05;

‡ *P* < 0.01;

§ *P* < 0.001 for incidence rate ratios vs. reference cohort.

The “other chromosomal anomalies” group had a 7-fold increased risk compared to the reference cohort. The recorded cancers included lymphocytic lymphoma, nephroblastoma, and pilocytic astrocytoma, in children with Wolf-Hirschhorn/4p- syndrome, Cri-du-chat/5p- syndrome, and Turner syndrome.

### Effect of Clinical Case Review and Classification

Because of earlier reports of increased cancer risk in children with hydrocephalus or heart defects, we evaluated these associations before and after clinical case review. Before clinical case review we had identified 15 cancer diagnoses in children with hydrocephalus, suggesting a 10-fold increased cancer risk (IRR, 10.7). However, with clinical case review, all but one of the hydrocephalus diagnoses were found to be secondary to a brain tumor obstructing cerebrospinal flow or to intraventricular hemorrhage in a preterm baby (1 case). After excluding these cases, the excess cancer risk disappeared (IRR 0.9, Table 3). Similarly, after the hierarchical classification of heart defects, many of the previously significant increased risks (tricuspid atresia/stenosis and atrioventricular septal defects) were reduced to levels comparable to the reference cohort, although a modestly elevated cancer risk remained for some septal defects (Table 3).

### Time Course of Cancer Risk

**Figure 2:**
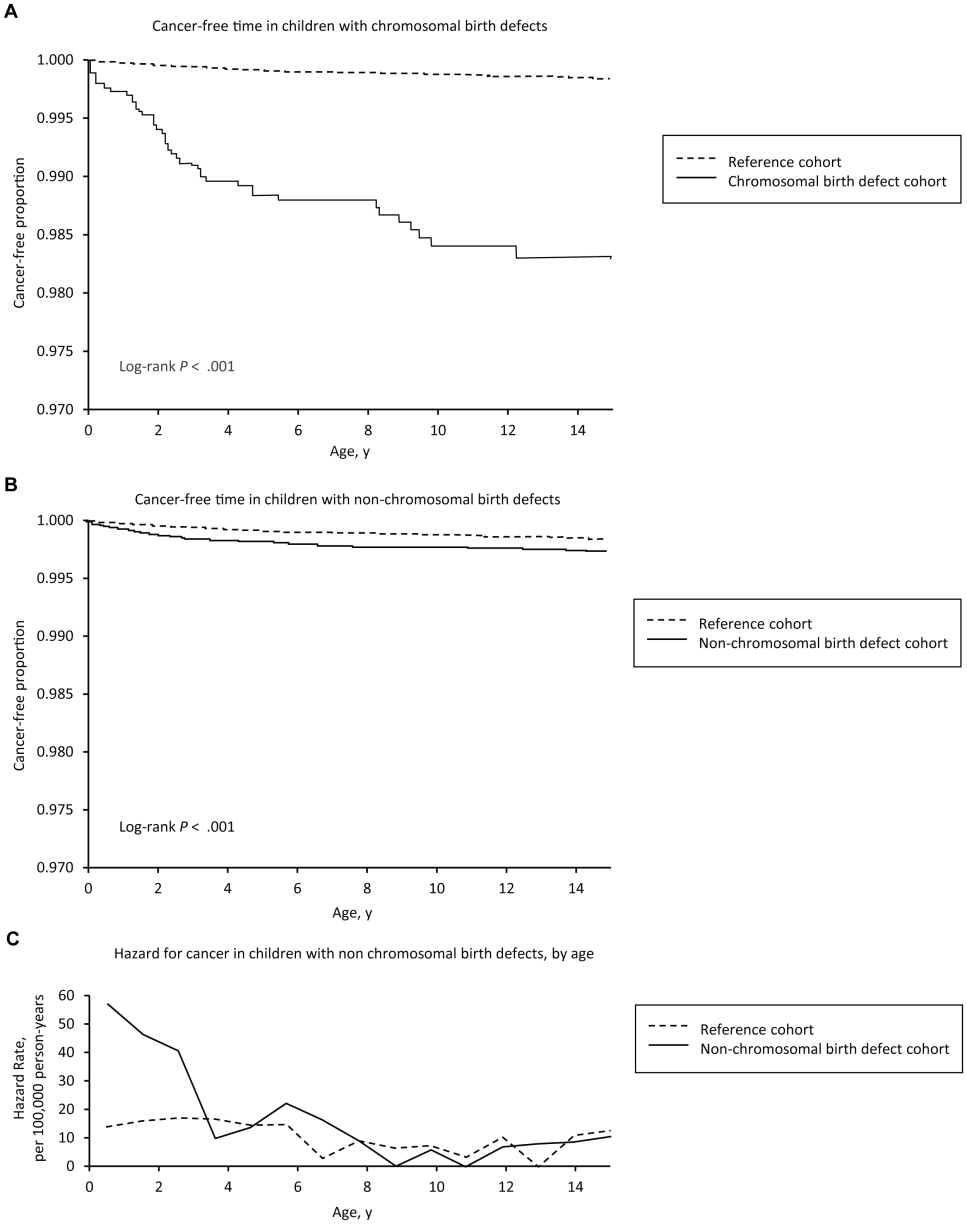
Cancer-free time curves of reference cohort vs. children with chromosomal anomalies (top panel) and children with non-chromosomal birth defects (middle panel), together with hazard rate for cancer by age in reference cohort vs. children with non-chromosomal birth defects (lower panel).

**Figure 3:**
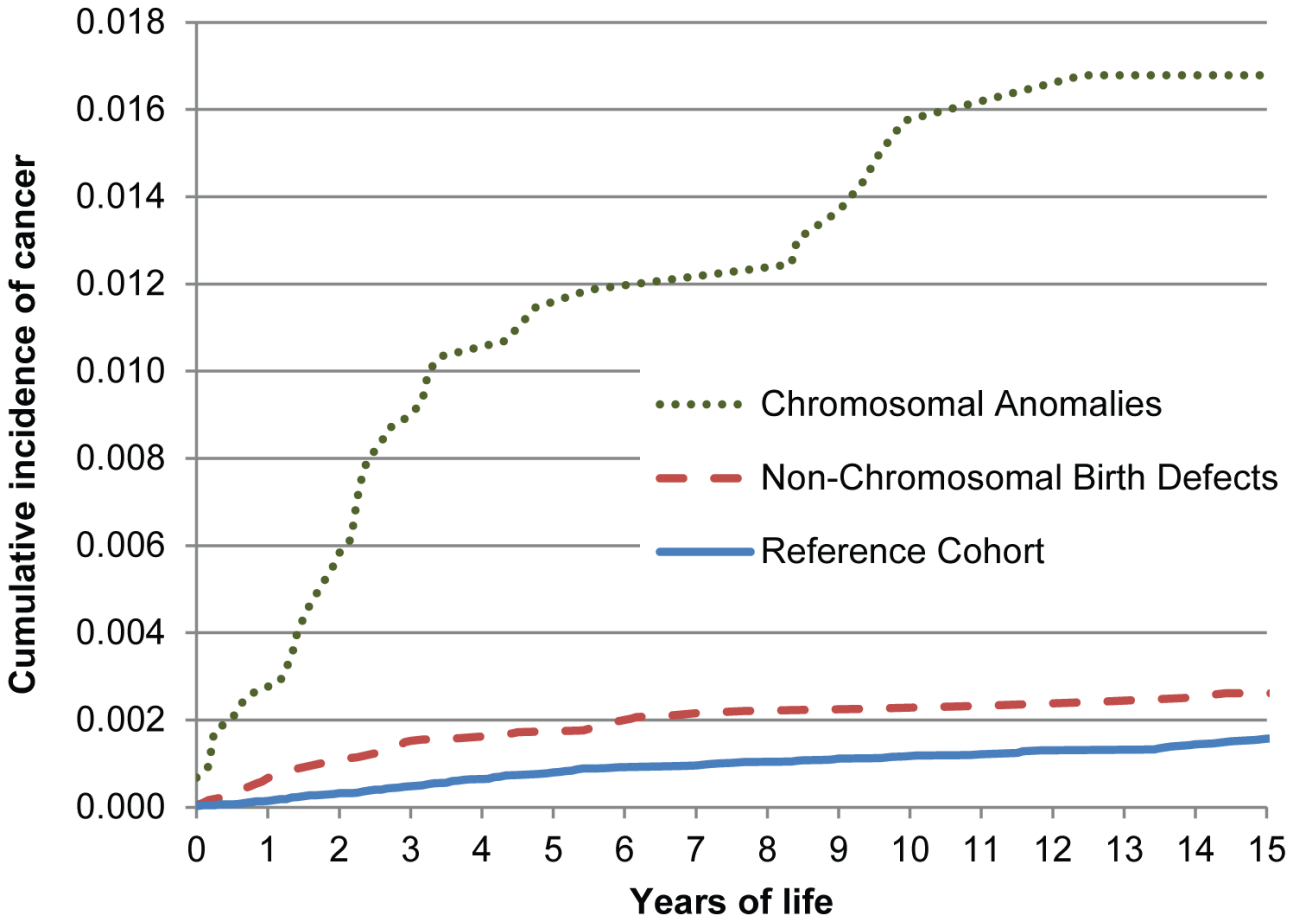
Cumulative incidence of cancer prior to age 15 years in children with non-chromosomal birth defects and with chromosomal anomalies, UTAZIA study. Cumulative incidence is expressed as a proportion (0.01 = 1 percent).

The cancer-free survival curves of the index and reference cohorts diverged significantly (p<0.001) soon after birth, markedly in the group with chromosomal anomalies (Figure 2, top panel), and less so in the group with non-chromosomal birth defects (Figure 2, middle panel). The hazard for cancer was highest in the first 3 to 5 years (Figure 1, lower panel), after which it was similar to the reference cohort (see Table S2 for additional data). A similar pattern was also seen among children with Down syndrome (data not shown). The corresponding cumulative incidence of cancer over time is shown in Figure 3.

## Discussion

### Key Results

In this population-based study, children and young adolescents with major non-chromosomal birth defects had a two-fold increased risk for cancer prior to age 15 years. However, cancer risk varied markedly by type of birth defect. In particular, we found increased cancer risks in children with microphthalmia, genitourinary defects (renal hypoplasia and obstructive renal defects), microcephaly, and cleft palate (Table 3). These associations could be clues to underlying cancer-predisposing conditions, which could be pursued in further studies. Conversely, and reassuringly, for many common defects the risk for cancer was low and similar to that observed in the reference population of children without birth defects.

With regard to cancer type, the excess cancer risk in children with non-chromosomal birth defects appeared to be driven mainly by tumors other than leukemias and lymphomas; such “embryonal” tumors, which include neuroblastoma, hepatoblastoma, and nephroblastoma, were temporally restricted to childhood and exceedingly rare later in life. Biologically, these tumors could represent the endpoint of mis-programmed organ development rather than the result of early exposure to carcinogens, and unlike typical cancers of the adult, may share pathophysiologic features with birth defects. To date, evidence of causal or mechanistic commonalities between birth defects and cancer has been scarce. Aside from a few Mendelian conditions (e.g. Fanconi syndrome [21]) and genomic disorders (e.g., Beckwith Wiedemann syndrome [20]), few genetic factors are known be associated both with major structural birth defects and childhood cancer. Some morphologic factors, such as low birth weight, are associated with birth defects and possibly with hepatoblastoma [30]; however, the causal role of low birth weight is unclear, as it could also represent an associated outcome or a marker of exposure (for example to parental smoking). Even less is known for environmental factors. For example, parental smoking is an established risk factor for orofacial clefts [31], but its link with most types of childhood cancers remains uncertain [32]–[34]. Finally, we found a 14-fold increased cancer risk among children with Down syndrome mainly due to lymphoid and myeloid leukemia. Although such relative risk is high, the absolute risk was fairly low. For example, for childhood leukemia, the absolute risk was in the order of 1 event in 600 person-years. Of note, in Down syndrome the risk for other types of cancers (e.g., the “embryonal” tumors) was not decreased, unlike what has been reported for cancers occurring later in the life of people with Down syndrome [35].

Cancer risk was also time-dependent. The cancer-free time curves of the reference cohort and the affected cohorts (Figure 2) diverge early in life. Interestingly, the curves continue to diverge for the children with chromosomal anomalies but seem to stabilize in early childhood for children with non-chromosomal birth defects. This is reflected in rising cumulative incidence curves in the main study cohorts (Figure 3), and is confirmed by the age-specific hazard rates (Figure 2, bottom panel) indicating an excess risk clustered in the first 3 to 5 years of life. The clinical implication is that clinical surveillance can be focused in time as well as by defect type.

Several high-quality studies, some recent, have examined aspects of the relation between birth defects and cancer; the findings, however, are variable and inconsistent [7]–[19], [36]–[48]. Among genitourinary defects, we confirmed an increased cancer risk in children with renal hypoplasia and obstructive renal defects [7], [8], even after careful case review to exclude hydronephrosis due to a mass effect of an abdominal tumor, but found no increased risk in children with hypospadias. Among children in the reference cohort, the incidence of cancer was higher in younger children, as has been seen repeatedly in large population surveys [7], [49], [50], and was very similar (11.7 vs. 11.6 per 100,000 PY) to that reported in a recent comparable Canadian study in the same age group [7].

Cancer risk was reassuringly low and comparable to the reference cohort for many common defects such as hypospadias, cleft lip (with or without cleft palate), pyloric stenosis, and hydrocephalus. This is at variance with some prior findings [8]–[10], [14], [15], [48]. The incorporation of clinical record review in our study could explain some of the discrepancies. For example, some studies reported an association between brain tumors and brain malformations such as hydrocephalus [8], [13], [14]. Concern has been raised [8] that in registry-based studies a diagnosis of hydrocephalus could be assigned to cases in which the hydrocephalus was secondary to a brain tumor obstructing the flow of cerebrospinal fluid, generating a spurious association. Because of this concern, we compared the association between brain cancer and hydrocephalus before and after clinical case review, and found that what appeared initially to be a strong association disappeared completely once cases of secondary hydrocephalus were identified and excluded.

For Down syndrome, prior studies [7]–[10], [12], [13], [17], [51]–[53] reported similar risk patterns, with some variation in the magnitude of the estimates. Children with trisomy 13 or 18 had a different risk profile. Previous case reports have documented the occurrence of Wilms tumor and hepatoblastoma in a few children with trisomy 18 [45], [54]–[56]. In our population-based cohort study we found no reported cases of cancer in this group, suggesting that an excess risk for cancer in children with trisomy 13 or 18, if present, is probably low and has a limited population impact; this limited impact is also due to the high mortality (and therefore short time at risk for cancer) in this group of children. This is consistent with an estimate suggesting that the absolute risk is in the order of 1% or less [56].

Compared to the studies reported to date, the current study adds information on specific birth defect groups that are clinically relevant and more precise than groupings based exclusively on the ICD coding conventions [7], [8]. Whereas the specific grouping may in some cases lead to relatively small sample sizes, it provides a level of detail that can be helpful to clinicians and researchers, and facilitates future meta-analyses.

### Limitations

In studies of birth defects, ascertainment is a significant challenge. In this study, which is based on liveborn infants followed over time, the rates of individual birth defects were consistent with prevalence rates in livebirths reported in high quality surveillance programs in the United States [22] and internationally [57]. Clinical case review was completed through review of medical records abstracted into birth defect and cancer surveillance reports, rather than direct clinical examination; thus, some diagnoses and clinical syndromes may have been missed.

An ascertainment bias of cancer in children with birth defects is unlikely, for several reasons. Children with birth defects are not monitored more closely for cancer than other children, and because cancer often develops years after a diagnosis of a major birth defect is made, the diagnostic evaluation for birth defects would not uncover cases of cancer. The converse, however, is probably a greater concern; finding previously undiagnosed birth defects during the diagnostic evaluation of a child with cancer. Factors that we think make such bias unlikely in this study include the focus on major birth defects, rather than minor anomalies that are more likely to have been missed early in life, and the clinical case review that examined the timing of diagnosis (to ensure that the birth defect diagnosis preceded that of the cancer). The case review also specifically mitigated another potential bias, that is, the miscoding as a birth defect of a structural finding that was a consequence of the cancer: one example of such miscoding is “hydrocephalus” secondary to a brain tumor impeding the circulation of cerebrospinal fluid.

We used population-based cohorts and population-based cancer and death registries to minimize selection and follow-up biases. Because no single cancer registry exists country wide, people who moved from the state of birth to another state and there developed cancer would not have been identified in the study. If this occurred differentially in the two cohorts, bias could have arisen. If similar in the different cohorts, such inaccuracies would tend to dilute the estimates of cancer risk towards the null. This study also had limited power to detect weak cancer risks associated with rare defects, a limitation common to many cohort studies. A meta-analysis of similar studies could strengthen the quantification of risk associated with rare defects. Several birth defect subgroups were small with few cancer events (Table 3) and the associated risk estimates are inherently unstable: the addition of a single or a few cases of cancer would change the risk estimates considerably. Nevertheless, the primary results–cancer risk among non-chromosomal birth defects and selected chromosomal anomalies–as well as the secondary analyses of the larger birth defect groups, were considerably more stable (Table 3), due to the comparatively large cohort size. Because of the many groups analyzed (<150 in Tables 3, 4, 5), chance may have played a role in some of the findings beyond the primary analyses. Correcting for multiple testing is controversial in epidemiologic studies; highly regarded epidemiologists disagree not only on methods but also on its intrinsic value [58]–[60]. Corrections are often applied in studies with a massive number of tests. Thousands of ‘tests’ are commonly done in genome-wide association studies or in nutrient studies with hundreds of compounds and multiple outcomes. In this study, however, the number of tests is still relatively low and the full scope of the analysis is provided, allowing for a direct evaluation of the potential role of chance. In addition, the analysis of the main groups (e.g., overall risk for cancer, with or without chromosomal anomalies) was hypothesis-driven and based on previous reports. Ultimately, however, as for all studies, associations will be confirmed or dismissed based on replication, and specifically, the joint findings of high quality studies.

Finally, the study focused on childhood cancer, defined as cancer occurring prior to age 15 years. Whether children with birth defects are at increased risk for cancer at a later age is an important question that can be answered with longer follow-up.

### Interpretation and Conclusions

We found a modest but significant increased risk for cancer for specific non-chromosomal birth defects, including microcephaly, congenital cataract, microphthalmia, cleft palate, renal hypoplasia, obstructive renal defects, and selected heart defects. Conversely, for many common birth defects, the risk was not significantly increased. In a clinical setting, focusing on overall risk estimates would be inaccurate and potentially misleading, and may produce unnecessary concern in many families with affected children while potentially failing to properly consider cancer risk in children with specific clinical phenotypes. In some of these clinical presentations associated with increased cancer risk, in-depth genetic consultation could be indicated, with the goal of identifying recognized cancer-predisposing conditions. Furthermore, with the increasing power of molecular evaluation, such clinical research could lead to discovering new conditions that could shed further light on the origin and development of cancers among children and adolescents.

## Supporting Information

Table S1 **Adjusted Relative Risk for cancer by major class of birth defects, UTAZIA study.**
(DOCX)

Table S2 **Hazard rate with 95% confidence interval (95%CI) for cancer, by age, in children with non-chromosomal birth defects and in reference cohort of children without birth defects, UTAZIA study.** Hazard rate is by 100,000 person-years.
(DOCX)

## References

[1] Hoyert DL, Mathews TJ, Menacker F, Strobino DM, Guyer B (2006) Annual summary of vital statistics: 2004. Pediatrics 117: 168–183. 16396875 10.1542/peds.2005-2587

[2] Centers for Disease Control and Prevention (2008) Update on Overall Prevalence of Major Birth Defects –- Atlanta, Georgia, 1978–2005. Morb Mortal Wkly Rep (MMWR) 57: 1–5. 18185492

[3] Christianson A, Howson CP, Modell B (2006) March of Dimes global report on birth defects: the hidden toll of dying and disabled children. White Plains, New York: March of Dimes Birth Defects Foundation.

[4] (2010) Resolutions of the 63rd World Assembly. World Health Organization.

[5] Rosano A, Botto LD, Botting B, Mastroiacovo P (2000) Infant mortality and congenital anomalies from 1950 to 1994: an international perspective. J Epidemiol Community Health 54: 660–666. 10942444 10.1136/jech.54.9.660 PMC1731756

[6] Yoon PW, Olney RS, Khoury MJ, Sappenfield WM, Chavez GF, et al (1997) Contribution of birth defects and genetic diseases to pediatric hospitalizations. A population-based study. Arch Pediatr Adolesc Med 151: 1096–1103. 9369870 10.1001/archpedi.1997.02170480026004

[7] Agha MM, Williams JI, Marrett L, To T, Zipursky A, et al (2005) Congenital abnormalities and childhood cancer. Cancer 103: 1939–1948. 15770693 10.1002/cncr.20985

[8] Bjorge T, Cnattingius S, Lie RT, Tretli S, Engeland A (2008) Cancer risk in children with birth defects and in their families: a population based cohort study of 5.2 million children from Norway and Sweden. Cancer Epidemiol Biomarkers Prev 17: 500–506. 18296646 10.1158/1055-9965.EPI-07-2630

[9] Mili F, Khoury MJ, Flanders WD, Greenberg RS (1993) Risk of childhood cancer for infants with birth defects. I. A record-linkage study, Atlanta, Georgia, 1968–1988. Am J Epidemiol 137: 629–638. 8470664 10.1093/oxfordjournals.aje.a116720

[10] Mili F, Lynch CF, Khoury MJ, Flanders WD, Edmonds LD (1993) Risk of childhood cancer for infants with birth defects. II. A record-linkage study, Iowa, 1983–1989. Am J Epidemiol 137: 639–644. 8470665 10.1093/oxfordjournals.aje.a116721

[11] Narod SA, Hawkins MM, Robertson CM, Stiller CA (1997) Congenital anomalies and childhood cancer in Great Britain. Am J Hum Genet 60: 474–485. 9042906 PMC1712528

[12] Rankin J, Silf KA, Pearce MS, Parker L, Ward Platt M (2008) Congenital anomaly and childhood cancer: A population-based, record linkage study. Pediatr Blood Cancer 51: 608–612. 18623214 10.1002/pbc.21682

[13] Windham GC, Bjerkedal T, Langmark F (1985) A population-based study of cancer incidence in twins and in children with congenital malformations or low birth weight, Norway, 1967–1980. Am J Epidemiol 121: 49–56. 3155484 10.1093/oxfordjournals.aje.a113982

[14] Altmann AE, Halliday JL, Giles GG (1998) Associations between congenital malformations and childhood cancer. A register-based case-control study. Br J Cancer 78: 1244–1249. 9820188 10.1038/bjc.1998.662 PMC2062998

[15] Bille C, Winther JF, Bautz A, Murray JC, Olsen J, et al (2005) Cancer risk in persons with oral cleft–a population-based study of 8,093 cases. Am J Epidemiol 161: 1047–1055. 15901625 10.1093/aje/kwi132 PMC2839121

[16] Infante-Rivard C, Amre DK (2001) Congenital anomalies in children with acute lymphoblastic leukaemia and in their family. Int J Epidemiol 30: 350–352. 11369741 10.1093/ije/30.2.350

[17] Linabery AM, Blair CK, Gamis AS, Olshan AF, Heerema NA, et al (2008) Congenital abnormalities and acute leukemia among children with Down syndrome: a Children’s Oncology Group study. Cancer Epidemiol Biomarkers Prev 17: 2572–2577. 18829445 10.1158/1055-9965.EPI-08-0284 PMC2610427

[18] Mertens AC, Wen W, Davies SM, Steinbuch M, Buckley JD, et al (1998) Congenital abnormalities in children with acute leukemia: a report from the Children’s Cancer Group. J Pediatr 133: 617–623. 9821417 10.1016/s0022-3476(98)70100-3

[19] Carozza SE, Langlois PH, Miller EA, Canfield M (2012) Are children with birth defects at higher risk of childhood cancers? Am J Epidemiol 175: 1217–1224. 22534203 10.1093/aje/kwr470

[20] Weksberg R, Shuman C, Beckwith JB (2010) Beckwith-Wiedemann syndrome. Eur J Hum Genet 18: 8–14. 19550435 10.1038/ejhg.2009.106 PMC2987155

[21] Auerbach AD (2009) Fanconi anemia and its diagnosis. Mutat Res 668: 4–10. 19622403 10.1016/j.mrfmmm.2009.01.013 PMC2742943

[22] National Birth Defects Prevention Network (2011) Selected Birth Defects Data from Population-based Birth Defects Surveillance Programs in the United States, 2004–2008 Birth Defects Res A Clin Mol Teratol. 91: 1028–1149.

[23] Abla O, Egeler RM, Weitzman S (2010) Langerhans cell histiocytosis: Current concepts and treatments. Cancer Treat Rev 36: 354–359. 20188480 10.1016/j.ctrv.2010.02.012

[24] Egeler RM, van Halteren AG, Hogendoorn PC, Laman JD, Leenen PJ (2010) Langerhans cell histiocytosis: fascinating dynamics of the dendritic cell-macrophage lineage. Immunol Rev 234: 213–232. 20193021 10.1111/j.0105-2896.2009.00883.x

[25] Botto LD, Lin AE, Riehle-Colarusso T, Malik S, Correa A (2007) Seeking causes: Classifying and evaluating congenital heart defects in etiologic studies. Birth Defects Res A Clin Mol Teratol 79: 714–727. 17729292 10.1002/bdra.20403

[26] Clark EB (1996) Pathogenetic mechanisms of congenital cardiovascular malformations revisited. Semin Perinatol 20: 465–472. 9090774 10.1016/s0146-0005(96)80062-0

[27] Clark EB (2001) Etiology of Congenital Cardiovascular Malformations: Epidemiology and Genetics. In: Allen HD, Gutgesell HP, Clark EB, Driscoll DJ, editors. Moss and Adams’ Heart Disease in Infants, Children, and Adolescents. Sixth ed. Philadelphia: Lippincott Williams & Wilkins. 64–79.

[28] Steliarova-Foucher E, Stiller C, Lacour B, Kaatsch P (2005) International Classification of Childhood Cancer, third edition. Cancer 103: 1457–1467. 15712273 10.1002/cncr.20910

[29] Romitti PA, Watanabe-Galloway S, Budelier WT, Lynch CF, Puzhankara S, et al (2010) Identification of Iowa live births in the Agricultural Health Study. Arch Environ Occup Health 65: 154–162. 20705576 10.1080/19338241003730903 PMC2936500

[30] Spector LG, Birch J (2012) The epidemiology of hepatoblastoma. Pediatr Blood Cancer 59: 776–779. 22692949 10.1002/pbc.24215

[31] Little J, Cardy A, Munger RG (2004) Tobacco smoking and oral clefts: a meta-analysis. Bull World Health Organ 82: 213–218. 15112010 PMC2585921

[32] Antonopoulos CN, Sergentanis TN, Papadopoulou C, Andrie E, Dessypris N, et al (2011) Maternal smoking during pregnancy and childhood lymphoma: a meta-analysis. Int J Cancer 129: 2694–2703. 21225624 10.1002/ijc.25929

[33] Klimentopoulou A, Antonopoulos CN, Papadopoulou C, Kanavidis P, Tourvas AD, et al (2012) Maternal smoking during pregnancy and risk for childhood leukemia: a nationwide case-control study in Greece and meta-analysis. Pediatr Blood Cancer 58: 344–351. 21990018 10.1002/pbc.23347

[34] Milne E, Greenop KR, Scott RJ, Ashton LJ, Cohn RJ, et al (2013) Parental smoking and risk of childhood brain tumors. Int J Cancer 133: 253–259. 23280760 10.1002/ijc.28004

[35] Yang Q, Rasmussen SA, Friedman JM (2002) Mortality associated with Down’s syndrome in the USA from 1983 to 1997: a population-based study. Lancet 359: 1019–1025. 11937181 10.1016/s0140-6736(02)08092-3

[36] Citak FE, Citak EC, Akkaya E, Kosan B, Ezer U, et al (2011) Minor anomalies in children with hematological malignancies. Pediatr Blood Cancer 56: 258–261. 20860040 10.1002/pbc.22689

[37] Durmaz A, Durmaz B, Kadioglu B, Aksoylar S, Karapinar D, et al (2011) The Association of minor congenital anomalies and childhood cancer. Pediatr Blood Cancer 56: 1098–1102. 21360657 10.1002/pbc.23049

[38] Green DM, Fiorello A, Zevon MA, Hall B, Seigelstein N (1997) Birth defects and childhood cancer in offspring of survivors of childhood cancer. Arch Pediatr Adolesc Med 151: 379–383. 9111437 10.1001/archpedi.1997.02170410053007

[39] Hartley AL, Birch JM, Blair V, Kelsey AM (1994) Malformations in children with soft tissue sarcoma and in their parents and siblings. Paediatr Perinat Epidemiol 8: 423–432. 7870626 10.1111/j.1365-3016.1994.tb00481.x

[40] Johnson KJ, Roesler MA, Linabery AM, Hilden JM, Davies SM, et al (2010) Infant leukemia and congenital abnormalities: a Children’s Oncology Group study. Pediatr Blood Cancer 55: 95–99. 20486175 10.1002/pbc.22495 PMC2904947

[41] Mallol-Mesnard N, Menegaux F, Lacour B, Hartmann O, Frappaz D, et al (2008) Birth characteristics and childhood malignant central nervous sytem tumors: the ESCALE study (French Society for Childhood Cancer). Cancer Detect Prev 32: 79–86. 18396378 10.1016/j.cdp.2008.02.003

[42] Mehes K, Kajtar P, Sandor G, Scheel-Walter M, Niethammer D (1998) Excess of mild errors of morphogenesis in childhood lymphoblastic leukemia. Am J Med Genet 75: 22–27. 9450852

[43] Merks JH, Ozgen HM, Koster J, Zwinderman AH, Caron HN, et al (2008) Prevalence and patterns of morphological abnormalities in patients with childhood cancer. JAMA 299: 61–69. 18167407 10.1001/jama.2007.66

[44] Nishi M, Miyake H, Takeda T, Hatae Y (2000) Congenital malformations and childhood cancer. Med Pediatr Oncol 34: 250–254. 10742060 10.1002/(sici)1096-911x(200004)34:4<250::aid-mpo3>3.0.co;2-w

[45] Olson JM, Hamilton A, Breslow NE (1995) Non-11p constitutional chromosome abnormalities in Wilms’ tumor patients. Med Pediatr Oncol 24: 305–309. 7700182 10.1002/mpo.2950240507

[46] Powell JE, Kelly AM, Parkes SE, Cole TR, Mann JR (1995) Cancer and congenital abnormalities in Asian children: a population-based study from the West Midlands. Br J Cancer 72: 1563–1569. 8519679 10.1038/bjc.1995.549 PMC2034071

[47] Little J (1999) Epidemiology of childhood cancer. IARC Scientific Publications 149: 306–317. Lyon (France): International Agency for Research on Cancer.

[48] Fisher PG, Reynolds P, Von Behren J, Carmichael SL, Rasmussen SA, et al.. (2012) Cancer in Children with Nonchromosomal Birth Defects. J Pediatr. 10.1016/j.jpeds.2011.12.006 PMC4490790 22244463

[49] Linet MS, Ries LA, Smith MA, Tarone RE, Devesa SS (1999) Cancer surveillance series: recent trends in childhood cancer incidence and mortality in the United States. J Natl Cancer Inst 91: 1051–1058. 10379968 10.1093/jnci/91.12.1051

[50] Parkin DM, Stiller CA, Draper GJ, Bieber CA (1988) The international incidence of childhood cancer. Int J Cancer 42: 511–520. 3170025 10.1002/ijc.2910420408

[51] Patja K, Pukkala E, Sund R, Iivanainen M, Kaski M (2006) Cancer incidence of persons with Down syndrome in Finland: a population-based study. Int J Cancer 118: 1769–1772. 16231334 10.1002/ijc.21518

[52] Sullivan SG, Hussain R, Glasson EJ, Bittles AH (2007) The profile and incidence of cancer in Down syndrome. J Intellect Disabil Res 51: 228–231. 17300418 10.1111/j.1365-2788.2006.00862.x

[53] Tabares-Seisdedos R, Dumont N, Baudot A, Valderas JM, Climent J, et al (2011) No paradox, no progress: inverse cancer comorbidity in people with other complex diseases. Lancet Oncol 12: 604–608. 21498115 10.1016/S1470-2045(11)70041-9

[54] Anderson CE, Punnett HH, Huff V, de Chadarevian JP (2003) Characterization of a Wilms tumor in a 9-year-old girl with trisomy 18. Am J Med Genet A 121A: 52–55. 12900902 10.1002/ajmg.a.20141

[55] Faucette KJ, Carey JC, Lemons RL, Toledano S (1991) Trisomy 18 and Wilms tumor: is there an association? Clin Res: 96A.

[56] Carey JC (2010) Trisomy 18 and trisomy 13 syndrome. In: Cassidy SB, Allanson JE, editors. Management of Genetic Syndromes. 3rd ed. Hoboken, New Jersey: Wiley-Blackwell. 807–823.

[57] International Clearinghouse for Birth Defects Surveillance and Research (2010) Annual Report 2010. In: Mastroiacovo P, editor. Rome Italy. Available: www.icbdsr.org. Accessed 2013 June 17.

[58] Greenland S (2008) Multiple comparisons and association selection in general epidemiology. Int J Epidemiol 37: 430–434. 18453632 10.1093/ije/dyn064

[59] Rothman KJ (1990) No adjustments are needed for multiple comparisons. Epidemiology 1: 43–46. 2081237

[60] Thompson JR (1998) Invited commentary: Re: “Multiple comparisons and related issues in the interpretation of epidemiologic data”. Am J Epidemiol 147: 801–806. 9583708 10.1093/oxfordjournals.aje.a009530

