# Supplementary material for: Cancer Risk in Children and Adolescents with Birth Defects: A Population-Based Cohort Study
Source: PLoS One. 2013 Jul 17;8(7):e69077. doi: 10.1371/journal.pone.0069077 (PMC3714243; doi:10.1371/journal.pone.0069077)
Supplement: Table S1 — Adjusted Relative Risk for cancer by major class of birth defects, UTAZIA study. (DOCX) [file pone.0069077.s001.docx]

**Table S1.** Adjusted Relative Risk for cancer by major class of birth defects, UTAZIA study.

| Diagnosis | **Cohort size** | **Person Years follow-up** | **Cases of cancer** | **Estimated Relative Risk** | | **95% CI** |
| --- | --- | --- | --- | --- | --- | --- |
| Cohort with Birth Defects | 44,151 | 363,659 | 123 | 3.0 | † | 2.3-4.0 |
| Non-chromosomal birth defects | 39,726 | 333,782 | 77 | 2.0 | † | 1.4-2.8 |
| Chromosomal anomalies | 4,425 | 29,877 | 46 | 14.8 | † | 9.9-22.1 |
| Trisomy 21 (Down syndrome) | 3,202 | 25,876 | 43 | 16.4 | † | 10.9-25.1 |

Note: risk estimates are adjusted for birth weight, gestational age, maternal age, maternal education, maternal race, and state, using Proportional Hazard models.

UTAZIA: Utah, Arizona, Iowa

† *P* < 0.05 for estimated relative risk (vs. reference cohort)
