# Supplementary material for: Cancer Risk in Children and Adolescents with Birth Defects: A Population-Based Cohort Study
Source: PLoS One. 2013 Jul 17;8(7):e69077. doi: 10.1371/journal.pone.0069077 (PMC3714243; doi:10.1371/journal.pone.0069077)
Supplement: Table S2 — Hazard rate with 95% confidence interval (95%CI) for cancer, by age, in children with non-chromosomal birth defects and in reference cohort of children without birth defects, UTAZIA study. Hazard rate is by 100,000 person-years. (DOCX) [file pone.0069077.s002.docx]

**Table S2.** Hazard rate with 95% confidence interval (95%CI) for cancer, by age, in children with non-chromosomal birth defects and in reference cohort of children without birth defects, UTAZIA study. Hazard rate is by 100,000 person-years.

|  | **Cohort with non-chromosomal birth defects** | |  | **Cohort without Birth Defects (Reference)** | |
| --- | --- | --- | --- | --- | --- |
| **Years** | **Hazard Rate** | **95% CI** |  | **Hazard Rate** | **95% CI** |
| 1 | 57.6 | 33.6-81.7 |  | 14.2 | 8.1-20.3 |
| 2 | 47.7 | 25-70.4 |  | 16.0 | 9.4-22.5 |
| 3 | 42.0 | 20-64.1 |  | 16.9 | 10-23.9 |
| 4 | 9.7 | 0-20.7 |  | 16.6 | 9.5-23.6 |
| 5 | 13.9 | 0.3-27.6 |  | 14.4 | 7.5-21.2 |
| 6 | 22.8 | 4.6-41.1.7 |  | 14.7 | 7.5-21.8 |
| 7 | 16.8 | 0.3-33.3 |  | 3.0 | 0-6.4 |
| 8 | 9.3 | 0-22.2 |  | 8.9 | 2.7-15 |
| 9 | 0.0 | - |  | 6.1 | 0.8-11.5 |
| 10 | 5.8 | 0-17.3 |  | 6.9 | 0.8-12.9 |
| 11 | 0.0 | - |  | 3.1 | 0-7.4 |
| 12 | 7.3 | 0-21.7 |  | 10.4 | 2.1-18.7 |
| 13 | 8.2 | 0-24.2 |  | 0.0 | - |
| 14 | 9.2 | 0-27.1 |  | 10.9 | 1.3-20.4 |
